# Chikungunya virus infection in *Aedes aegypti* is modulated by L-cysteine, taurine, hypotaurine and glutathione metabolism

**DOI:** 10.1371/journal.pntd.0011280

**Published:** 2023-05-02

**Authors:** Ankit Kumar, Jatin Shrinet, Sujatha Sunil

**Affiliations:** Vector Borne Diseases Group, International Centre for Genetic Engineering and Biotechnology, New Delhi, India; Beijing Children’s Hospital Capital Medical University, CHINA

## Abstract

**Background:**

Blood meal and infections cause redox imbalance and oxidative damage in mosquitoes which triggers the mosquito’s system to produce antioxidants in response to increased oxidative stress. Important pathways activated owing to redox imbalance include taurine, hypotaurine and glutathione metabolism. The present study was undertaken to evaluate the role of these pathways during chikungunya virus (CHIKV) infection in *Aedes aegypti* mosquitoes.

**Methodology:**

Using a dietary L-cysteine supplement system, we upregulated these pathways and evaluated oxidative damage and oxidative stress response upon CHIKV infection using protein carbonylation and GST assays. Further, using a dsRNA based approach, we silenced some of the genes involved in synthesis and transport of taurine and hypotaurine and then evaluated the impact of these genes on CHIKV infection and redox biology in the mosquitoes.

**Conclusions:**

We report that CHIKV infection exerts oxidative stress in the *A*. *aegypti*, leading to oxidative damage and as a response, an elevated GST activity was observed. It was also observed that dietary L-cysteine treatment restricted CHIKV infection in *A*. *aegypti* mosquitoes. This L-cysteine mediated CHIKV inhibition was coincided by enhanced GST activity that further resulted in reduced oxidative damage during the infection. We also report that silencing of genes involved in synthesis of taurine and hypotaurine modulates CHIKV infection and redox biology of *Aedes* mosquitoes during the infection.

## Author summary

Mosquitoes need human blood for the development of their eggs. During this process, when the mosquito bites human already harboring viruses such as chikungunya virus (CHIKV), the virus is taken up by the mosquito, which carries the virus for the rest of its life and transmits the virus to healthy individuals during subsequent blood meals. However, the impact of these viruses on the mosquito physiology is poorly understood. This study is an attempt to evaluate the impact of CHIKV infection on the redox biology of *Aedes* mosquitoes. Oxidative stress is a critical response to several biological activities and is controlled by a vast network of pathways of genes/proteins. Some of these pathways are L-cysteine centric, such as taurine, hypotaurine and glutathione metabolism. By providing dietary supplements of L-cysteine and by reducing the expression of some of the genes in the above mentioned pathways, we studied the role of these pathways in regulating oxidative stress during CHIKV infection. Our results suggest that dietary supplement of L-cysteine and selected genes of the taurine/hypotaurine and glutathione pathways was found to control oxidative damage in the mosquitoes during CHIKV infection by regulating the expression of Glutathione-s-transferases (GST) enzyme, a well known antioxidant molecule.

## Introduction

Oxidative stress reflects on an abnormal production of reactive oxygen species (ROS) creating a hostile environment in that system thereby causing cellular damage by affecting membranes, lipids, proteins, and nucleic acids. For instance, protein carbonylation is a major detrimental outcome of increased ROS and causes irreversible damage to the proteins, produced by oxidation in the side chains of amino acids [1–3]. Another harmful effect of spiked ROS is lipid peroxidation which targets molecules like glycolipids, phospholipids, and cholesterol resulting in disruption of membranes and many other cellular functions [4,5]. Additionally, byproducts of lipid peroxidation are also involved in the enhancement of protein carbonylation [4,6]. A homeostasis between production and neutralization of ROS termed redox homeostasis is a vital phenomenon employed by the cell to combat oxidative stress [7,8].

Oxidative stress in mosquitoes is generated by various factors such as blood meal, infections, and abiotic agents like insecticides and leads to enhanced generation of reactive oxygen species (ROS), protein carbonylation and lipid peroxidation [9,10]. In response to this oxidative stress, the mosquito’s immune system responds by inducing antioxidants like catalase, Glutathione-s-transferases (GSTs), and superoxide dismutases (SODs) that aid in restoring redox homeostasis during cellular metabolism [9–13]. Arboviruses like dengue and zika viruses are known to induce oxidative stress in mosquitoes; at the same time, oxidative stress in turn plays a key role in modulating the infection of these viruses in *Aedes* mosquitoes [13,14]. Mosquitoes have their own defense mechanism to fight the infections, such as melanization, generation of antimicrobial peptides (AMPs), CEC-like peptides, RNAi, and other systemic antiviral strategies [15]. Oxidative stress is a major mechanism that is employed by the mosquito’s system to overcome viral infection [9,13,14,16,17]

**Fig 1:**
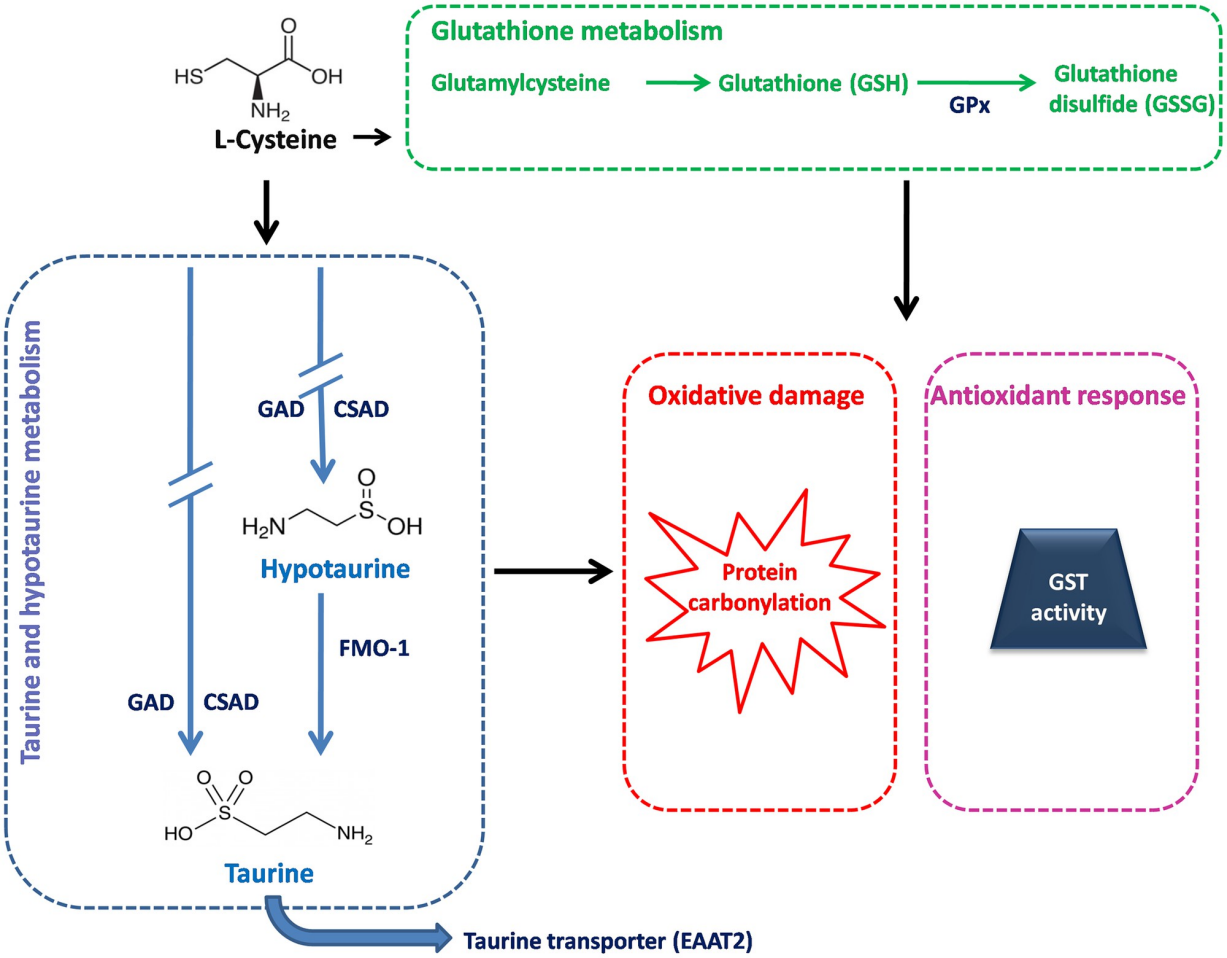
Schematic representation of the pathways and the genes targeted in the study. Figure depicts the molecules of taurine/hypotaurine metabolism (blue dotted box) and glutathione metabolism (green dotted box) that were targeted and the readout of the outcome upon their modulation. In taurine and hypotaurine metabolism, GAD and CSAD, molecules that are responsible for the synthesis of both taurine and hypotaurine, and FMO1 that is involved in the synthesis of taurine from hypotaurine were selected. Block arrow represents taurine transportation aided by EAAT2, a taurine transporter. Discontinuous blue arrows represent intermediate components of taurine and hypotaurine synthesis. Blue arrow represents the direct reaction involved in hypotaurine to taurine mediated by FMO-1. In glutathione metabolism, the reaction of glutamylcysteine conversion to glutathione, followed by Gpx mediated production of glutathione disulfide is represented as green arrows. All the five genes of interest have been represented in dark blue text. Protein carbonylation was studied as a marker of oxidative damage and GST activity was evaluated for measuring the mosquito’s antioxidant response to CHIKV infection, L-cysteine dietary supplementation and the loss-of-function assays. Protein carbonylation has been shown as a subset of oxidative damage inside the dotted red box. A dark blue trapezoid inside a dotted pink box represents GST activity shown as a subset of antioxidant response.

One of our previous studies indicated that CHIKV infection caused a significant differential expression of taurine and hypotaurine metabolism pathway in infected *A*. *aegypti* mosquitoes [18]. Our study further divulged that L-cysteine was significantly downregulated during CHIKV infection. The present study was undertaken to delve deeper into the role of L-cysteine and taurine/hypotaurine in maintaining redox homeostasis during CHIKV infection. L-cysteine is an important precursor molecule involved in the synthesis of both taurine and hypotaurine, and three enzymes, namely, glutamate decarboxylase (GAD), cysteine sulfinic acid decarboxylase (CSAD), and flavin-containing monooxygenase 1 (FMO1) assist in the final stages of the synthesis of hypotaurine and/or taurine. Apart from this, L-cysteine is also known to regulate glutathione synthesis [19,20], and enzymes such as glutathione peroxidases (Gpx) and Glutathione transferases (GSTs) are known to play critical role in maintaining redox homeostasis, by metabolizing the byproducts of lipid peroxidation and controlling protein carbonylation [21–23]. By providing surplus L-cysteine as a dietary supplement and by silencing some of the critical molecules using dsRNA treatment, we evaluated redox biology during CHIKV infection in *A*. *aegypti* mosquitoes (Fig 1).

## Material and method

### Mosquitoes

Mosquitoes used in this study were laboratory-adapted *A*. *aegypti* (F8-13) previously collected from wild populations in New Delhi, India. Mosquito lines were maintained in the in-house facility at 26–28°C, with 55–80% relative humidity and 12:12 light: dark diurnal cycle. For maintenance mosquitoes were fed 10% sucrose solution and mice blood *ad libitum*.

### Virus

CHIKV used in this study (Accession no: JF950631.1) was a clinical isolate collected during 2010 outbreak from New Delhi, India [24]. Virus was cultured alternatively in C6/36 and Vero cells to maintain the natural course of infection. Virus used to infect the mosquitoes was cultured in Vero cells with DMEM, 2% fetal bovine serum, penicillin and streptomycin at 37°C with 75% relative humidity. Viral stocks were prepared 42 hours post infection and stored at -80°C. Standard plaque assay protocol was used to quantify the viral titer [25].

### Blood feeding and nanoinjections

Artificial blood feeding was performed using water-jacketed glass feeders supplied with continuous supply of water maintained at 37°C. A mixture of 70% defibrinated blood, 10% FBS and 20% DMEM was used as blood meal. In case of infected groups, this mixture contained 10^6^ plaque forming units (PFU) per mL of CHIKV. Mosquitoes starved for atleast 6 hours were allowed to feed through the feeders for approximately 1 hour, followed by separation of fully gorged female mosquitoes for further experiments [25]. These mosquitoes were used to estimate protein carbonylation and GST activity.

CHIKV was also introduced in the mosquitoes through intrathoracic nanoinjections using Nanoject II (Drummond). 69nL of virus culture containing 4×10^6^ PFU/mL of CHIKV was injected in cold anesthetized female mosquitoes [26].

### Estimation of protein carbonylation

Protein carbonylation was estimated using 2, 4-dinitrophenylhydrazine (DNPH) derivitization method [27–29]. 50uL of total protein concentration matched mosquito extracts were added to 50uL of 10mM DNPH in clear and flat bottom 96 well plates. After 10 minutes, 25uL of 6M NaOH was added to each well and the plates were incubated for 10 minutes at room temperature. Then absorbance was read at 450nm using Spectromax2 (Molecular Devices, USA).

### GST activity quantification

GST activity in protein extracts was measured using Glutathione-S-Transferase assay kit CS0410 (Sigma-Aldrich, USA), using manufacturer’s protocol. 20uL of protein concentration matched samples were added to the different wells in 96 well flat clear bottom plate, followed by addition of 180uL substrate solution containing 200mM L-glutathione, 100mM 1-chloro-2,4-dinitrobenzene (CDNB) and phosphate buffer saline. Immediately upon addition of substrate solution to the samples, absorbance was read every minute for 30 minutes at 340nm, using Spectromax 2. Total GST activity was derived using the formula given with the kit.

### dsRNA preparation for knockdown in mosquitoes

RNA was extracted from mosquitoes using trizol method and templates for in vitro transcription were prepared using the primers listed in S1 Table and PrimeScript one-step RT PCR kit (Takara Bio, Japan). The PCR product was gel purified and used to perform in vitro transcription using Megascript T7 Transcription kit AM1334 (Thermo Scientific, USA) following the manufacturer’s instructions. GFP-C1 (Clontech- TAKARA Bio, Japan) was used as a template for the amplification of control dsRNA, targeting eGFP.

### Climbing assay

To assess mosquito’s fitness upon gene knockdown using dsRNA mediated silencing, we performed climbing assay on the treated mosquitoes as per published protocols [30] with some modifications. Group of dsRNA injected mosquitoes were kept in a container of length 6cm and diameter 15cm. After 48 hours these mosquitoes were gently tapped to the bottom of the containers. Mosquitoes crossing a median line at 4cm of height in 60 seconds were counted and were considered climbing assay qualified.

### RNA isolation and qRT-PCR

RNA isolation from individual mosquito whole bodies was performed using Trizol method. Virus genome equivalents were quantified by performing one-step qRT-PCR (QuantiTect SYBR green qRT-PCR kit, Qiagen, Germany) using CHIKV specific primers. Absolute quantification method of qRT-PCR was used for CHIKV genome equivalent estimation. For gene expression analysis in silencing experiments, comparative analysis method of qRT-PCR was performed using gene specific primers. RPS17 was used as housekeeping gene for data normalization. Details of all the primers are available in S2 Table.

### Data analysis

GraphPad prism 6 for windows was used to perform statistical analysis. The type of statistical tests performed for each data has been mentioned in their respective legends. Asterisks represent significant differences. *P* < 0.05 was defined as the significance for all the tests.

## Results

### Blood meal and CHIKV infection induce oxidative stress in *A*. *aegypti*

One of the fundamental functions in female mosquito physiology is blood feeding which has a direct impact on its life cycle that incidentally opens up avenues for harboring pathogens in the process [15,31]. However, blood feed *per se* may result in mounting oxidative stress to the mosquitoes. In order to deduce the level of oxidative stress blood feeding induces in mosquitoes, we estimated GST activity and protein carbonylation during artificial blood feeding using blood with and without CHIKV. Comparison was made between the sucrose-fed mosquitoes, mosquitoes fed with uninfected blood and those fed with CHIKV spiked blood. We used sodium arsenite (NaAsO_2_), an inorganic compound known to induce oxidative damage in insects and other model systems, as a positive control, to estimate a scale of perturbation in protein carbonylation and GST specific activity by different treatments in the mosquitoes. In this group, the cotton pads soaked with 10% sucrose solution that was used for sugar feeding the mosquitoes was spiked with 50μM NaAsO_2_ The concentration to be used for the experiments was decided after testing groups of mosquitoes with 100uM and 50uM NaAsO2 based on previous published data [32]. At 100uM, we observed complete mortality within 48 hours whereas at 50uM, the mosquitoes survived for atleast 72 hours. Based on these results, concentration of 50uM of NaAsO2 was used for treatment to elicit oxidative damage.

**Fig 2:**
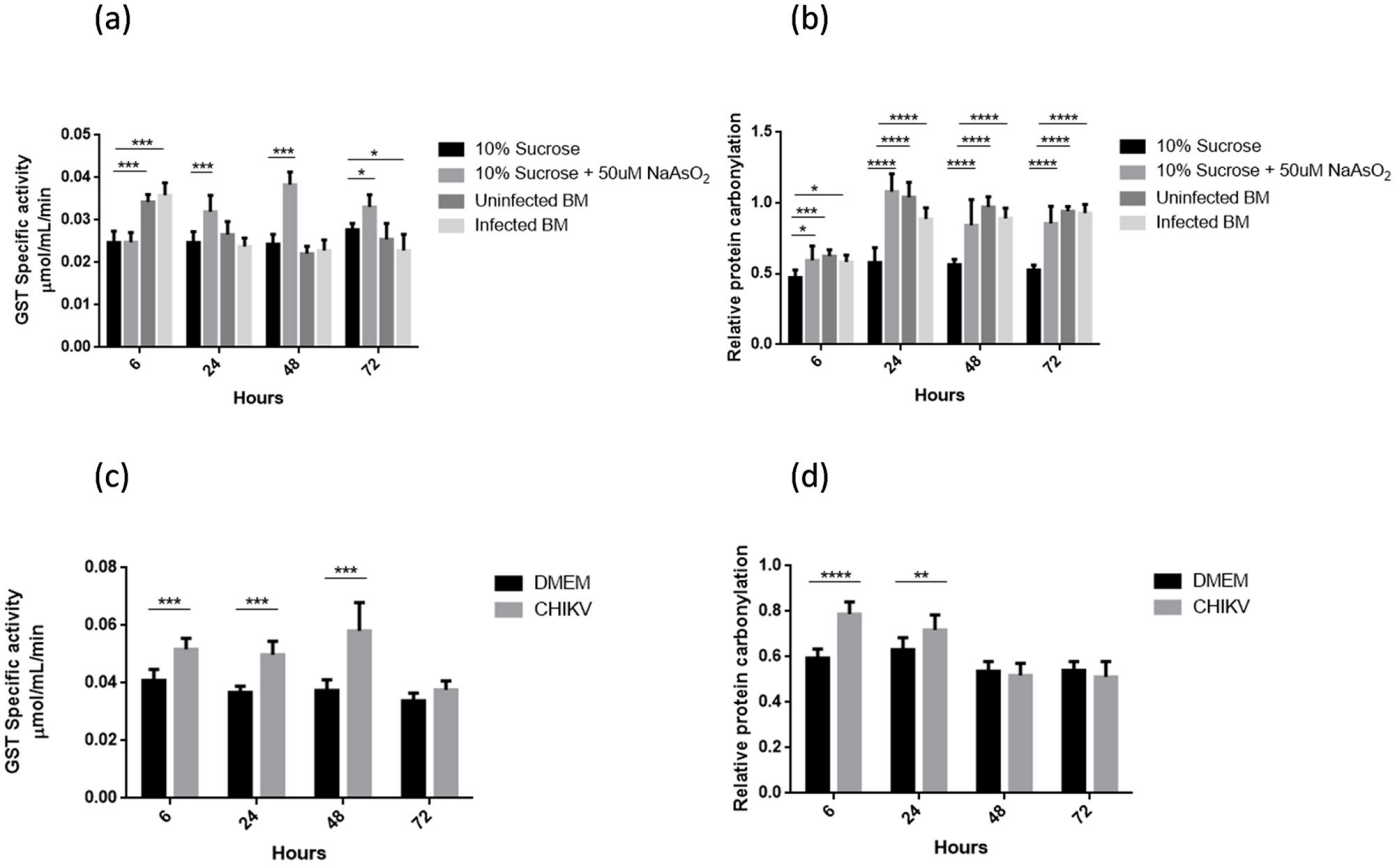
Oxidative stress upon CHIKV infection through blood meal and nanoinjections. (a-b). GST enzyme activity and relative protein carbonylation in mosquitoes upon blood meal and CHIKV spiked blood meal, compared with naive or 10% sucrose fed mosquitoes and mosquitoes receiving 10% sucrose spiked with NaAsO_2_ as diet. (c-d) GST enzyme activity and protein carbonylation in mosquitoes upon intrathoracic nanoinjections of 2% DMEM and CHIKV in 2% DMEM. Each sample was prepared by pooling 3 mosquito whole bodies from the respective time points, in RIPA lysis buffer followed by homogenization and stored at -80°C. A minimum of three such pooled samples was prepared at all the time-points from every experiment and each experiment was performed atleast thrice independently. Error bars represent standard deviation calculated using Two-way Anova (Tukey’s multiple comparison test).

All mosquitoes were harvested at specific time points, namely, 6 hrs, 24 hrs, 48 hrs, and 72 hrs post feeding/injections. Analyses of the various groups with the mosquitoes receiving 10% sucrose only, revealed that blood meal itself caused a significant change in oxidative stress levels in mosquitoes at early time points (Fig 2a and 2b). We observed that NaAsO_2_ treatment induced no significant change in GST specific activity at 6h post treatment, but exhibited highest elevation in the later time-points as compared to the other groups (Fig 2a). Highest GST specific activity was observed in NaAsO_2_ treated mosquitoes at 48h post treatment. On the other hand, protein carbonylation was recorded to be comparable in the mosquitoes receiving sodium arsenite and either of the blood meal, uninfected or infected. NaAsO_2_ treatment showed maximum oxidative damage at 24h post treatment, exhibiting ~100% increase in protein carbonylation (Fig 2b). Also, it was observed that GST specific activity and protein carbonylation were both affected significantly by infected or uninfected blood meal (Fig 2a and 2b). A striking observation was that GST specific activity was found to be ~ 40% enhanced upon blood meal only in the early 6 hour time point, whereas protein carbonylation was found to be almost doubled for a period of 3 days; the duration required by the mosquitoes to digest or clear the ingested blood (Fig 2a and 2b). These results suggest that GST activity is triggered transiently by the blood meal for a limited period of time, while oxidative damage was observed persistently for 3 days post blood meal. On the other hand, exposure to NaAsO_2_ generated prolonged antioxidant response when compared to the uninfected or infected blood meal, while the extent of oxidative damage caused by NaAsO_2_ was comparable with the mosquitoes receiving either of the blood meal. It was observed that mosquitoes receiving diet of 10% sucrose spiked with 50μM NaAsO_2_ did not survive CHIKV nanoinjections and showed complete mortality within 6 hours post infections. Also, it could be seen that the presence of CHIKV in blood meal had no significant change in GST activity or protein carbonylation, as compared to normal uninfected blood meal. This observation established that even if CHIKV infection caused any redox imbalance in the mosquitoes, blood meal mediated infection would not be an efficient method to study that change. Therefore it was necessary to use an alternate method of infecting mosquitoes. For this purpose, we resorted to intrathoracic nanoinjections to infect mosquitoes with CHIKV.

In case of nanoinjections, CHIKV culture was injected in the mosquitoes intrathoracically, and same volume of DMEM, which served as the control, was injected to another set of mosquitoes. Control group mosquitoes showed no significant change in GST activity upon nanoinjections over time, and remained comparable for the course of 72 hours (Fig 2c). Whereas atleast 20% enhancement in GST activity was observed during the initial 48 hours upon infection when compared to the mock DMEM injected group. And GST activity reached to the baseline levels by 72h post CHIKV infections as compared to DMEM injected control group. On the other hand protein carbonylation was elevated by ~23% at 6 hours post CHIKV infection, and subsequently reduced to ~15% at 24hpi. At 48hpi, the level further dipped and was comparable to that of the uninfected group (Fig 2d). DMEM injected mosquitoes served as controls in these experiments. Taken together, these results suggest that though the oxidative damage caused due to CHIKV infection in mosquitoes is not prolonged, the protective response of GST specific activity is enhanced upon infection probably for protection against oxidative damage (Fig 2c and 2d). These results further demonstrated that nanoinjections were a more efficient technique to study oxidative stress caused by arboviral infection in mosquitoes. Henceforward, we performed all experiments pertaining to CHIKV infection and its impact on oxidative stress using nanoinjections.

### Dietary L-cysteine restricts CHIKV infection and reduced oxidative stress in *A*. *aegypti*

**Fig 3:**
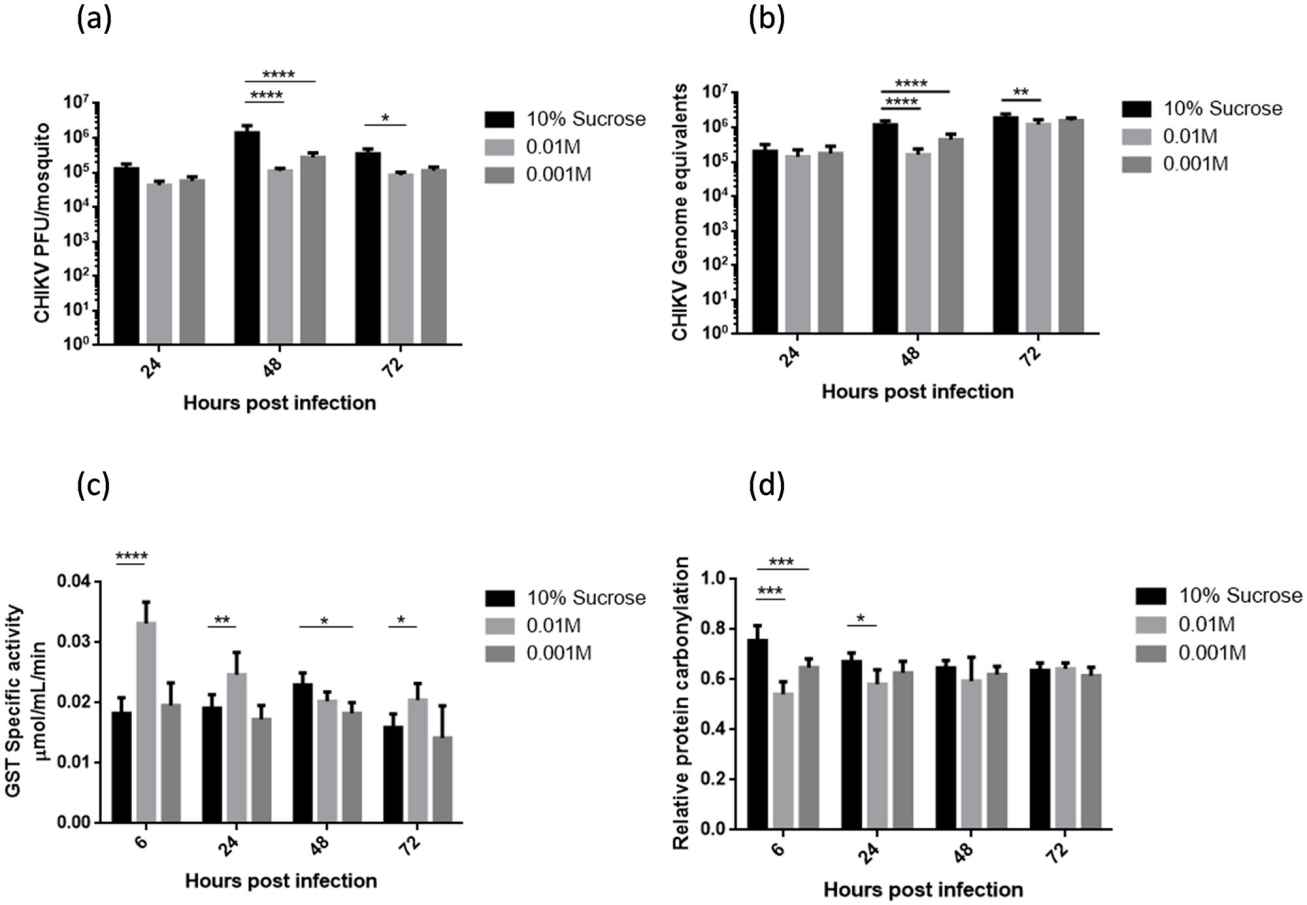
CHIKV kinetics and oxidative stress with dietary L-Cysteine supplement. Mosquitoes were exposed to L-cysteine rich diet for atleast 48 hours prior to CHIKV nanoinjections. L-cysteine was added to the sucrose solution supplied to the mosquitoes through cotton pads. (a) CHIKV titer determined using plaque assay performed from individual/ single mosquito whole body homogenized in 1mL of DMEM and filtered using 0.2 micron syringe filters. (b) CHIKV genome equivalents quantified using qRT-PCR on the RNA isolated from individual mosquito whole bodies. These experiments were performed atleast thrice and atleast 5 individual mosquitoes were taken at each time point from every independent experiment. (c and d) GST activity and relative protein carbonylation performed in the mosquito samples collected from the L-cysteine rich diet experiments. Pool of 3 mosquitoes was used to prepare protein extracts for these experiments. 3 pooled samples were taken from each experiment. Each experiment was performed atleast thrice independently. Error bars represent standard deviation and statistical significance was calculated using Two-way Anova (Dunnett’s multiple comparison test).

As indicated by our previous study, L-cysteine was found to be downregulated upon CHIKV infection [18]. Since L-cysteine is also a central molecule between taurine/hypotaurine metabolism and glutathione metabolism, L-cysteine treatment was used to study the association between these two metabolic pathways with CHIKV infection and oxidative stress. For this purpose, we sought to administer L-cysteine orally to the mosquitoes and then proceeded to evaluate its effect on taurine/hypotaurine and glutathione pathways. We created a dietary supplement comprising of 10% sucrose solution added with different concentrations (0.01M and 0.001M) of L-cysteine and used this as mosquito feed. Thus, 2–3 days old female mosquitoes were separated in three different cartons and supplied with sucrose solution supplemented with L-cysteine. After two days of exposure to dietary L-cysteine, CHIKV was injected into the thorax of these mosquitoes. In this experiment, mosquitoes that were exposed to 10% sucrose treatment were taken as control group. Upon completion of nanoinjections, mosquitoes were collected at different time points (6h, 24h, 48h, and 72h). These samples were then processed for plaque assays, GST activity assay and protein carbonylation estimation. Results showed that CHIKV infection was more than 50% inhibited by L-cysteine treatment in the individual mosquitoes in a dose-dependent manner (Fig 3a), indicating that L-cysteine could be a potential CHIKV inhibitor. We also validated this L-cysteine mediated inhibition of CHIKV using qRT-PCR to quantify viral genome equivalents in the mosquito RNA samples. We observed similar inhibition of CHIKV at 48 hpi in case of mosquitoes receiving L-cysteine. Also, unlike plaque based viral quantification we observed ~30% CHIKV inhibition by mean in mosquitoes receiving 0.01M L-cysteine as compared to the CHIKV only group (Fig 3b). Hence to validate its activity as a CHIKV inhibitor we performed inhibition assays in mosquito (Aag2) and mammalian (Vero) cell lines. Antiviral assays showed no CHIKV inhibition in both the cell lines, indicating that CHIKV is not directly targeted by L-cysteine, but there might be some other mechanism involved which is restricting CHIKV infection in mosquitoes upon the higher dosage of L-cysteine (S1 Fig).

On the other hand, results from GST activity assays provided a noticeable observation that GST activity was increased by atleast 25% during the initial 48hpi in the mosquitoes which received 0.01M L-cysteine supplement (Fig 3c). Also in the mosquitoes with enhanced GST activity protein carbonylation was found to be reduced in the early time points i.e., 6h and 24h, by ~25% and ~12% respectively. Taking all these findings together, it could be concluded that L-cysteine restricts CHIKV infection in the *A*. *aegypti* mosquitoes. And this CHIKV inhibition is possibly being regulated by enhanced GST specific activity observed in mosquitoes receiving L-cysteine rich diet. Also, it could be seen that enhanced GST activity is reducing the oxidative damage caused during CHIKV infection, as evident in Fig 3d.

### Association of taurine, hypotaurine and glutathione metabolism with oxidative stress during CHIKV infection in *A*. *aegypti*

Having established that L-cysteine played an important role in the regulation of oxidative stress in *A*. *aegypti* during CHIKV infection, we next sought to dissect the pathways associated with this molecule. Therefore in addition to L-cysteine, we evaluated the role of genes involved in the synthesis of taurine and hypotaurine, which are the major molecules affected by L-cysteine as it is involved in their metabolism. We knocked down some of the major enzymes involved in taurine, hypotaurine and glutathione metabolism, and evaluated their impact on CHIKV infection and oxidative stress. Based on earlier reports, glutamate decarboxylase (GAD), cysteine sulfinic acid decarboxylase (CSAD), flavin-containing monooxygenase 1 (FMO1) and excitatory amino acid transporter 2 (EAAT2) were selected for further evaluation. GAD and CSAD are known to be directly associated with taurine and hypotaurine synthesis, hence we hypothesized that loss-of-function assays of these molecules may provide us a model of depleted taurine and hypotaurine levels in mosquitoes [33–38]. FMO1 is another molecule reported to play a role further downstream in the pathway and was involved in the taurine synthesis from hypotaurine and was included in this study [39]. EAAT2 is an amino acid transporter that has been validated in the *Drosophila* model as a taurine transporter and was used as a molecule that upon silencing could block taurine transport. Alongside taurine/hypotaurine pathways, we targeted an important molecule, glutathione peroxidase (GPx), which is known to be essential in glutathione metabolism, another pathway associated with L-cysteine. Gpx is reported to be a regulator of oxidative stress and protects the organism from oxidative damage [22,23] and is known to inhibit lipid peroxidation. It is known that byproducts of lipid peroxidation are involved in protein carbonylation; therefore Gpx indirectly controls protein carbonylation as well. Based on these rationales, we evaluated its role in oxidative stress by estimating GST specific activity and protein carbonylation during CHIKV infection.

**Fig 4:**
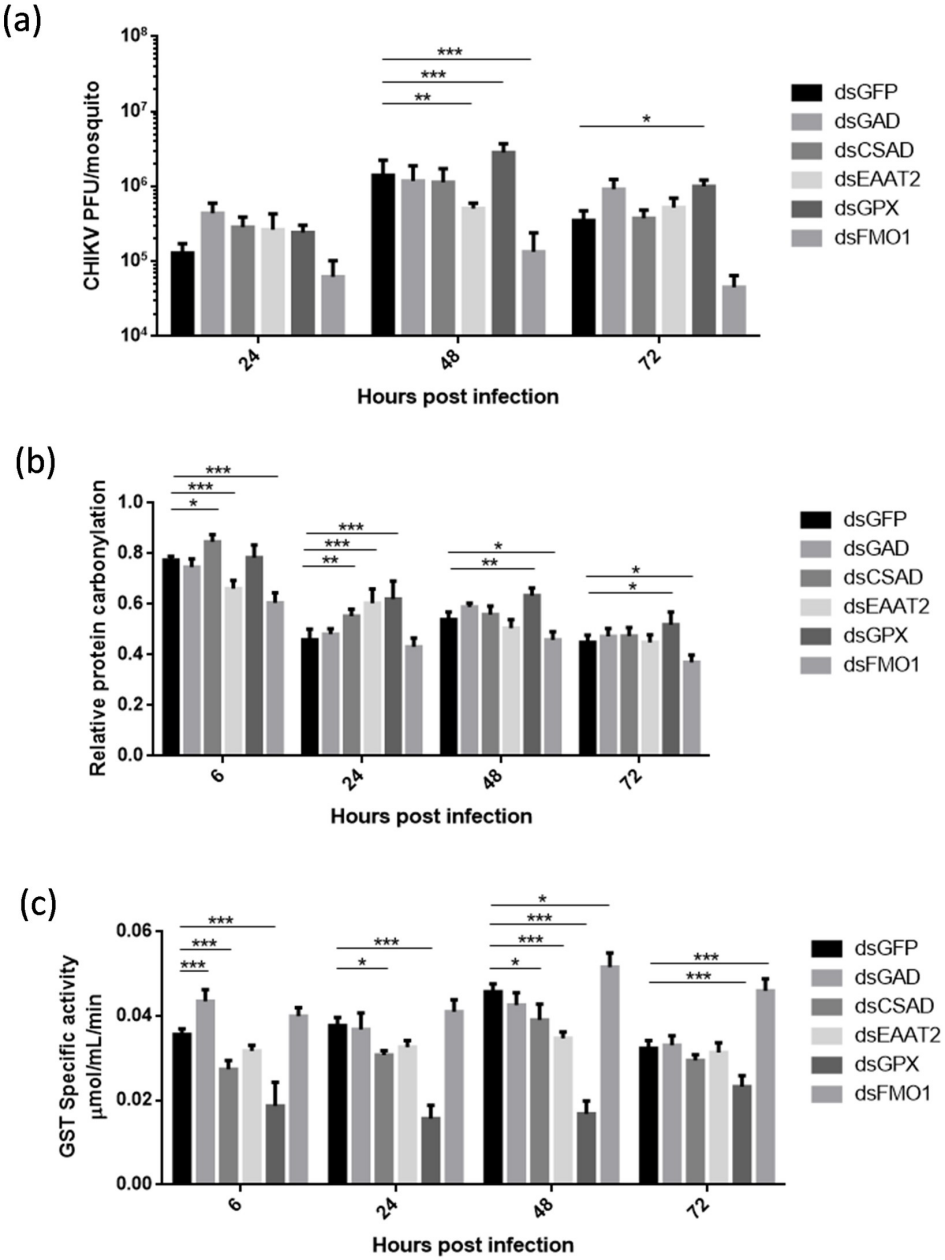
Impact of gene silencing on CHIKV infection and oxidative stress. Knockdown experiments were performed using dsRNA mediated gene silencing method. (a) CHIKV titer in the individual mosquitoes at the time points post CHIKV injections. CHIKV titers were evaluated in atleast 5 individual mosquitoes using plaque assay at every time-point in each experiment and every experiment was performed atleast thrice independently. (b and c) GST activity and relative protein carbonylation in mosquitoes infected with CHIKV upon gene knockdowns. Pool of 3 mosquitoes was used to prepare protein extracts in RIPA lysis buffer and atleast three such pools were collected at every time point. Each experiment was performed atleast thrice independently. Error bars represent standard deviation calculated using Two-way Anova (Tukey’s multiple comparison test).

Gene silencing was performed by injecting gene specific dsRNA in the mosquitoes. GFP dsRNA was used as a negative control in silencing experiments. Mosquitoes receiving 500ng of dsRNA (100ng in case of CSAD) intrathoracically showed maximum silencing. In this experiment, 500ng of dsRNA prepared using GFP sequence was used as a negative control for silencing, as dsGFP has no target site in *A*. *aegypti* (validated using NCBI BLAST). Gene knockdown was validated using qRT-PCR. Based on silencing data, CHIKV nanoinjections were performed in the gene-silenced mosquitoes to evaluate the impact of gene knockdown on CHIKV progression and redox homeostasis. After 48hrs post gene silencing, mosquitoes were subjected to climbing assay and only the mosquitoes qualifying the climbing assay (S3 Table) were infected intrathoracically with CHIKV and were observed for a period of three days. The extent of silencing, CHIKV titers, GST activity and protein carbonylation was estimated in the mosquitoes at 24 hour intervals and correlated with each other. Gene silencing at specific time-points was evaluated by qRT-PCR using the primers listed in S2 Table (data shown in S2 Fig). Loss-of-function assays revealed that out of the three enzymes, GAD silencing caused a minor increase in CHIKV titer at 72hpi as compared to the dsGFP control group, CSAD silencing showed no significant change in CHIKV titer. Interestingly, FMO1 silencing resulted in 85–90% reduction of CHIKV titer at 48hpi. With respect to the taurine transporter, EAAT2, a reduction of ~65% in viral titer was observed at 48hpi. Gpx silencing enhanced CHIKV titres to more than 50% after the initial infection time-points (Fig 4a).

We next sought to evaluate the role of taurine and hypotaurine in regulating oxidative damage that might be caused owing to CHIV infection. For this purpose, we evaluated the oxidative damage in the gene silenced mosquitoes post CHIKV infection using protein carbonylation assay. An ~20% increased protein carbonylation was observed in the dsCSAD group 24hpi, after which it reduced to the levels observed in the control, whereas dsGAD did not show any protein carbonylation throughout the observation duration. Silencing of FMO1 resulted in a significant decrease in protein carbonylation (p value 0.05–0.001) over the time points. A significant increase in protein carbonylation (p value 0.001) was observed in the dsEAAT2 group. Gpx silenced group showed increased protein carbonylation in all the time-points after 6hpi (Fig 4b).

Finally, as a measurement of oxidative stress response, we evaluated GST activity in the gene silenced groups. A ~30% increase in GST activity in the initial time-points (6h) was observed in the dsGAD injected mosquitoes, and subsequently the activity reduced to that of the control. In case of CSAD silenced mosquitoes, there was a gradual increase of GST activity until 48 hpi. FMO1 silenced mosquitoes exhibited significantly increased GST activity during the 72 hours of observation, with the activity peaking at 48 hpi. EAAT2 exhibited a reduced GST activity at 48hpi (~20%); whereas, as expected, upon Gpx silencing, there was more than 50% reduction in GST activity until 48hpi and a 30% reduction at 72 hpi (Fig 4c).

In order to infer the impact of CHIKV infection on oxidative damage and its response, we next correlated the above results. Of the genes involved in taurine synthesis and transport, FMO1 exhibited the maximum impact on CHIKV infection, oxidative damage and the response to damage. A positive correlation was observed between CHIKV titers and oxidative damage, whereas there was a negative correlation to GST activity in the FMO1 silenced group. In case of Gpx, we observed a similar correlation to that of FMO1, albeit in the opposite sense. Enhanced CHIKV titer was observed with increased oxidative damage and reduced GST activity.

## Discussion

Anautogeny is an essential phenomenon of mosquito’s reproductive cycle, as adult female mosquitoes need vertebrate blood’s meal for the maturation of its eggs. Consumption of blood triggers release of ovary ecdysteroidogenic hormone (OEH) and insulin like peptide-3 (ILP3), which stimulates maturation of eggs [40–42]. Though the consumption of blood is essential for mosquitoes, blood meal itself could trigger oxidative damage in the mosquitoes. Blood meal and redox state is also known to regulate the fecundity and insecticide resistance in the mosquitoes [43,44]. Even though not the primary objective of this study, our results established the effect of blood meal on redox homeostasis in mosquitoes, as we observed enhanced oxidative damage in mosquitoes at 24h and 48h post blood meal. Several studies have previously established that in order to maintain homeostasis, mosquitoes produce molecules and mechanisms that prevent oxidative damage caused by the consumption of blood. Such mechanisms include the release of peritropic matrix in the midgut [45] and also the production of antioxidant molecules like catalase, SODs, Gpx and GSTs, further preventing oxidative damage as a consequence of blood meal [13].

In addition to blood meal, oxidative stress is also triggered by various infections in the mosquitoes and induction of antioxidant response has been reported to play vital roles in the establishment of pathogens in mosquitoes [13,46]. For example, *Aedes* mosquitoes infected with *Wolbachia* show enhanced expression of genes involved in maintaining redox balance, and it is well established that *Wolbachia* infected mosquitoes develop resistance to arboviral infections like DENV, ZIKV and CHIKV [47–49]. *Wolbachia* infection induces ROS levels in the mosquitoes, which triggers the toll pathway that mediates the production of antimicrobial peptides (AMPs like defensins and cercopins). AMPs have been reported to inhibit DENV infection in *Aedes* mosquitoes [50]. Also, DENV infection in mosquito cells elevates ROS generation in the cells, in response to which GST activity is also increased in the infected cells. Reduction in GST activity leads to cell death in DENV infected mosquito cells [51]. Another study indicated that catalase controls oxidative damage in the midgut upon consumption of DENV infected blood meal by the *Aedes* mosquitoes, but it also increases infection prevalence in the mosquitoes [13]. The present study establishes that CHIKV infection in *A*. *aegypti* mosquitoes is regulated by antioxidant response, and that L-cysteine intake inhibits CHIKV infection, while controlling oxidative damage by regulating the GST activity.

L-cysteine has been previously known to regulate the synthesis of glutathione [20], and thereby participate in maintaining redox homeostasis through a cascade of biochemical reactions amongst which GSTs play a crucial role [10]. In mosquitoes, role of GSTs have been well studied in case of insecticides and has been reported as one of the major enzyme involved in the oxidative stress management induced by exposure to insecticides [10]. GSTs function by preventing and repairing the oxidative stress/ ROS induced damages by the activity of Gpx enzyme or by conjugation with HNE, a byproduct of lipid peroxidation [52], and the main rationale for us to include Gpx in the present study. Furthermore, since we established the association of GST activity with CHIKV infection and oxidative damage control through L-cysteine treatment, we attempted to decipher the role L-cysteine mediated pathways during CHIKV infection in mosquitoes.

Delving into the molecular mechanisms involved in maintaining redox homeostasis, we targeted specific transcripts involved in production and transportation of taurine respectively such as GAD, CSAD, FMO1 and EAAT2, along with Gpx [39,53], and observed that some of these genes modulated CHIKV infection in the mosquitoes. Specifically, out of three enzymes involved in taurine synthesis, namely GAD, CSAD and FMO1, only FMO1 was found to have a substantial impact over CHIKV infection and oxidative stress during the infection. We observed that FMO1 significantly promoted CHIKV infection over a period of time, coinciding with increased oxidative damage and a decreased oxidative stress response. In comparison, the taurine transporter, EAAT2 was more subtle in its impact; the increase in CHIKV titer was limited to a brief period and unlike FMO1, EAAT2 did not impact oxidative damage or stress response that significantly, suggesting a dynamic nature of the transporter in maintaining redox homeostasis during infection. In contrast to these molecules, on the other arm of L-cysteine mediated metabolism, Gpx played the opposite role, i.e., of increasing oxidative stress response and regulating CHIKV infection and a reduced oxidative damage.

**Fig 5:**
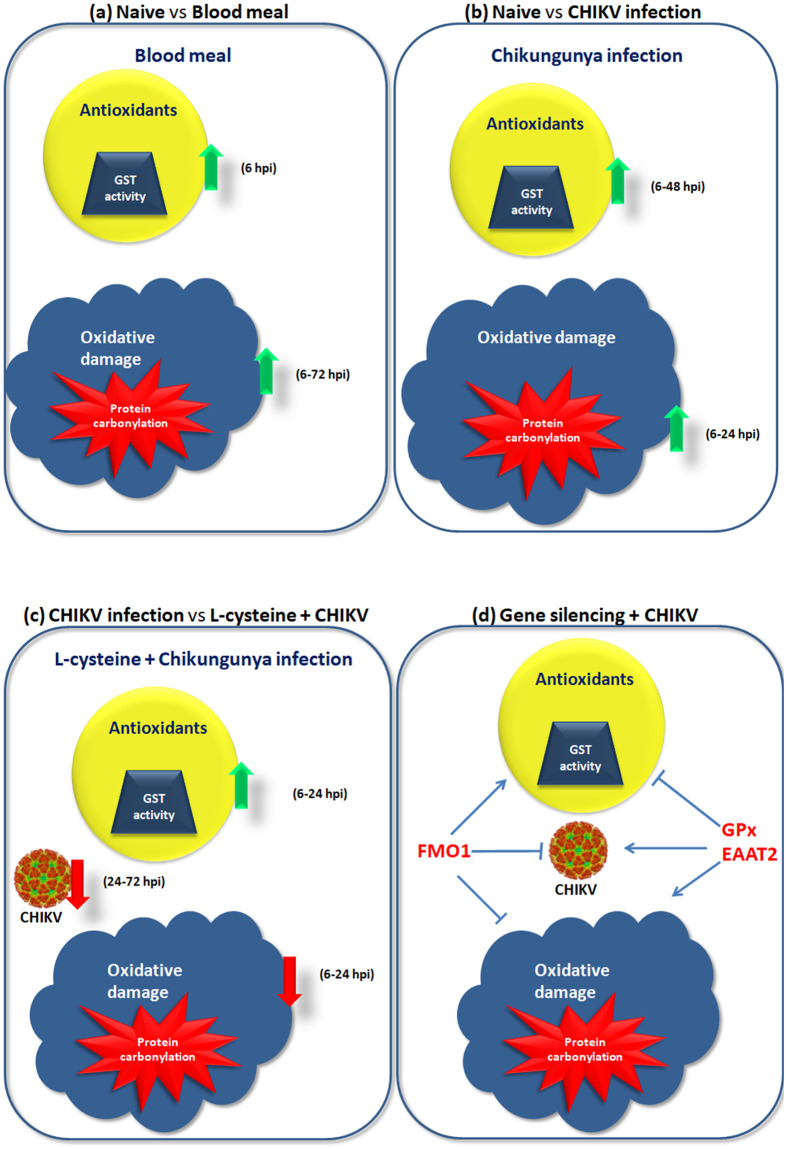
Overview of oxidative stress upon CHIKV infection in *Aedes* mosquitoes and role of L-cysteine during CHIKV infection. Blood meal clearly disrupts the redox balance in mosquitoes, by causing oxidative damage. Mosquito’s system responds to this by increasing the GST activity, which is a well known antioxidant. Secondly, intrathoracic CHIKV infection causes oxidative damage in the mosquitoes, coinciding with enhanced GST activity. Dietary L-cysteine supplement restricts the CHIKV infection in mosquitoes and elevates GST activity during the infection. L-cysteine rich diet during infection also showed reduced oxidative damage during CHIKV infection. Silencing of FMO1 gene resulted in elevated GST activity, coinciding with reduced protein carbonylation and CHIKV infection. While silencing of Gpx and EAAT2 showed exactly the opposite observation i.e., reduced GST activity coinciding with enhanced CHIKV infection and protein carbonylation.

Primarily, our study aimed to investigate the role of taurine, hypotaurine and glutathione metabolism in CHIKV infection in *Aedes* mosquitoes. In a previous study, it was reported that L-cysteine, which is a central molecule in taurine, hypotaurine and glutathione metabolism, is downregulated upon CHIKV infection in *A*. *aegypti* mosquitoes [18]. Since L-cysteine is a known precursor of glutathione, which is an antioxidant, we hypothesized this molecule could directly associate CHIKV infection and redox balance in *Aedes* mosquitoes. This study establishes three important aspects in *Aedes* biology during CHIKV infection (Fig 5). Firstly, blood meal imparts more significant oxidative damage to *Aedes* than CHIKV infection, but CHIKV elicits prolonged GST activity response as compared to blood meal. Secondly, dietary L-cysteine restricts CHIKV infection exclusively in the mosquitoes in a dose-dependent manner and controls oxidative damage by elevating the GST activity as early as in the initial 6–24 hours post infection. Thirdly, genes or enzymes involved in taurine and hypotaurine synthesis play a significant role in management of the oxidative stress in *A*. *aegypti* during CHIKV infection (Fig 5). Another observation made upon data analysis was that even minor changes in GST activity and protein carbonylation may impart physiologically significant difference on CHIKV infection and oxidative stress in mosquitoes. The impact of these minor variations in GST activity and protein carbonylation exhibiting physiologically relevant effect on different phenomena in other models have been also reported earlier [29,54,55].

Taken together, this study correlates viral infection with oxidative damage and GST activity; and the implication can be manifold. For instance, this aspect can be better utilized for studying the role of insecticides in controlling viral infections in the mosquitoes. Previous reports have already established the impact of insecticides on redox biology of mosquitoes. For example, DDT is known to affect the GSTs in mosquitoes and is also known to cause oxidative damage in the mosquito’s system. Hence based on the correlation of redox biology with CHIKV in our study it would be interesting to evaluate the effect of widely used insecticides on arboviruses like CHIKV in the mosquito system.

## Supporting information

S1 Fig CHIKV titer in Aag2 and Vero cell lines upon L-cysteine treatment. CHIKV infection kinetics in Aag2 and Vero cells was determined in presence of different non-toxic concentrations of L-cysteine. Infection kinetics was performed 12 well plates and media supernatant was harvested at the given time-points. CHIKV quantification was performed using standard plaque assay protocol on Vero cells in 96 well plates. Three replicates of each time-point were analyzed for CHIKV titer estimation in every experiment and each experiment was performed thrice independently. Statistical analysis was performed using two way anova.
(TIF)

S2 Fig Relative expression of genes upon CHIKV infection during the knockdown studies at specific time-points. dsRNA mediated silencing of selected genes was performed in separate groups of *A*. *aegypti* mosquitoes followed by CHIKV injections in the same mosquitoes. Relative expression analysis of genes was performed in their respective gene knockdown groups using one-step qRT-PCR with the primers listed in S2 Table. Atleast 5 individual mosquito whole bodies were examined for relative expression in each experiment and every experiment was performed atleast thrice independently. One way anova was performed for statistical analysis.
(TIF)

S1 Table Primers for gene specific dsRNA preparation used for gene silencing studies. (DOT)

S2 Table Primers used for qRT-PCR based gene expression analysis. (DOT)

S3 Table Climbing assay analysis post gene-silencing and CHIKV infection. (DOC)
