# Supplementary figures and images for: Chikungunya virus infection in Aedes aegypti is modulated by L-cysteine, taurine, hypotaurine and glutathione metabolism
Source: PLoS Negl Trop Dis. 2023 May 2;17(5):e0011280. doi: 10.1371/journal.pntd.0011280 (PMC10153688; doi:10.1371/journal.pntd.0011280)

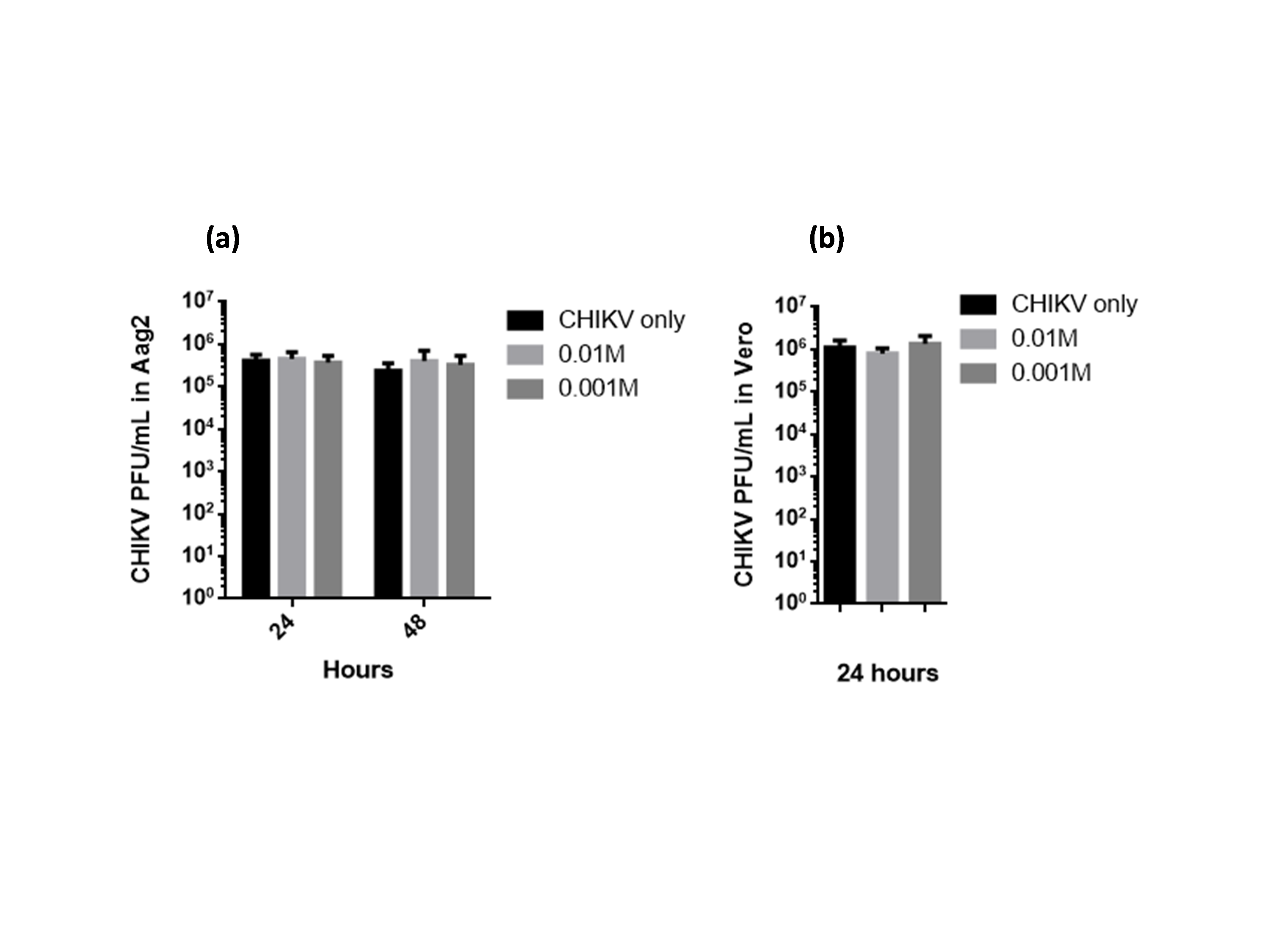

Supplement: S1 Fig — CHIKV infection kinetics in Aag2 and Vero cells was determined in presence of different non-toxic concentrations of L-cysteine. Infection kinetics was performed 12 well plates and media supernatant was harvested at the given time-points. CHIKV quantification was performed using standard plaque assay protocol on Vero cells in 96 well plates. Three replicates of each time-point were analyzed for CHIKV titer estimation in every experiment and each experiment was performed thrice independently. Statistical analysis was performed using two way anova. (TIF) [file pntd.0011280.s001.tif]
