# Supplementary material for: Chikungunya virus infection in Aedes aegypti is modulated by L-cysteine, taurine, hypotaurine and glutathione metabolism
Source: PLoS Negl Trop Dis. 2023 May 2;17(5):e0011280. doi: 10.1371/journal.pntd.0011280 (PMC10153688; doi:10.1371/journal.pntd.0011280)
Supplement: S1 Table — (DOT) [file pntd.0011280.s003.dot]

**Supplementary table 1: Primers for gene specific dsRNA preparation used for gene** silencing studies.

| **Target** | **Direction** | **Sequence** |
| --- | --- | --- |
| **GAD** | Forward | TAATACGACTCACTATAGGGATTCCGTTCTTCGTCAATGC |
|  | Reverse | TAATACGACTCACTATAGGGTTCGGCTAGCTCCATCAGTT |
| **CSAD** | Forward | TAATACGACTCACTATAGGGGATGGATGTTTGGGTGGAAC |
|  | Reverse | TAATACGACTCACTATAGGGTAAAACCAATCGGAACCCAG |
| **EAAT2** | Forward | TAATACGACTCACTATAGGGTCTAGGAGCCGTCTTCGGTA |
|  | Reverse | TAATACGACTCACTATAGGGCAATGTGACTAATCCGGCCT |
| **GPx** | Forward | TAATACGACTCACTATAGGGCAATGAACCTTCGTCGGTCT |
|  | Reverse | TAATACGACTCACTATAGGGCGTCCCCGTTGACGTATATC |
| **FMO1** | Forward | TAATACGACTCACTATAGGGCCAACTTGCCGAAGGAAATAATGGG |
|  | Reverse | TAATACGACTCACTATAGGGGAACGCACAGAGTTTGTGTAGCT |
