# Supplementary material for: Chikungunya virus infection in Aedes aegypti is modulated by L-cysteine, taurine, hypotaurine and glutathione metabolism
Source: PLoS Negl Trop Dis. 2023 May 2;17(5):e0011280. doi: 10.1371/journal.pntd.0011280 (PMC10153688; doi:10.1371/journal.pntd.0011280)
Supplement: S2 Table — (DOT) [file pntd.0011280.s004.dot]

**Supplementary table 2: Primers used for qRT-PCR based gene expression analysis.**

| **Target** | **Direction** | **Sequence** | |
| --- | --- | --- | --- |
| **GAD** | Forward | TGAAGCGAATCCGAGAGCAATCA | |
|  | Reverse | ACTGCATCATTCGAGATTTGATGATG | |
| **CSAD** | Forward | ATGATTCCGGATGAGTTGGCGAA | |
|  | Reverse | GACAGATGTCGGCAATTCGTCCAAAC | |
| **EAAT2** | Forward | TGGATACAGTTGGTCTACCAGCAGAAG | |
|  | Reverse | TTTGACTGTAGTGGGCGACAATGGC | |
| **GPx** | Forward | ATGTTCCTGTTGCGACTTCTAACCC | |
|  | Reverse | CAAAACGCTAGATGTTGAAAGCAGGTTTATC | |
| **FMO1** | Forward | ATGGTGAATGACATGATCAATGACAAGAAACG | |
|  | Reverse | CCGTATTCGTTCTTGTCCACCTCTTCG | |
| **CHIKV** | Forward | | TACCCATTTATGTGGGGC |
|  | Reverse | | GCCTTTGTACACCACGATT |
| **RPS17** | Forward | | GTGAGCGCAGAGACAACTAC |
|  | Reverse | | TCCAGCTGCTTCAACATCTC |
