# Supplementary material for: Chikungunya virus infection in Aedes aegypti is modulated by L-cysteine, taurine, hypotaurine and glutathione metabolism
Source: PLoS Negl Trop Dis. 2023 May 2;17(5):e0011280. doi: 10.1371/journal.pntd.0011280 (PMC10153688; doi:10.1371/journal.pntd.0011280)
Supplement: S3 Table — (DOC) [file pntd.0011280.s005.doc]

| **Groups** | **dsGFP** | **dsGAD** | **dsCSAD** | **dsEAAT2** | **dsGpx** | **dsFMO1** |
| --- | --- | --- | --- | --- | --- | --- |
| **Total no of mosquitoes injected with dsRNA** | 120 | 120 | 120 | 120 | 120 | 120 |
| **No of mosquitoes qualifying climbing assay post dsRNA injections** | 97 | 83 | 74 | 93 | 87 | 78 |
| **Total no of mosquitoes injected with CHIKV** | 97 | 83 | 74 | 93 | 87 | 78 |
| **No of mosquitoes qualifying climbing assay post CHIKV injections** | 75 | 66 | 63 | 76 | 68 | 58 |

**Supplementary table 3 – Details of mosquitoes qualifying the climbing assay test.**
